# Foliar transcriptomes reveal candidate genes for late blight resistance in cultivars of diploid potato *Solanum tuberosum* L. Andigenum Group

**DOI:** 10.3389/fpls.2023.1210046

**Published:** 2023-09-11

**Authors:** Germán De la Cruz, Raúl Blas, Willmer Pérez, Edgar Neyra, Rodomiro Ortiz

**Affiliations:** ^1^ Laboratorio de Genética y Biotecnología Vegetal, Facultad de Ciencias Agrarias, Universidad Nacional de San Cristóbal de Huamanga (UNSCH), Ayacucho, Peru; ^2^ Instituto de Biotecnologia (IBT), Facultad de Agronomia, Universidad Nacional Agraria La Molina (UNALM), Lima, Peru; ^3^ Plant Pathology Laboratory, Crop and Systems Sciences Division, International Potato Center, Lima, Peru; ^4^ Unidad de Genómica, Laboratorios de Investigación y Desarrollo, Facultad de Ciencias e Ingeniería, Universidad Peruana Cayetano Heredia, Lima, Peru; ^5^ Departamento Académico de Tecnología Médica, Facultad de Medicina, Universidad Peruana Cayetano Heredia, Lima, Peru; ^6^ Department of Plant Breeding, Swedish University of Agricultural Sciences, Lomma, Sweden

**Keywords:** gene expression, genome, host plant resistance, *Phytophthora infestans*, *Solanum tuberosum*

## Abstract

Characterization of major resistance (*R*) genes to late blight (LB) –caused by the oomycete *Phytophthora infestans–* is very important for potato breeding. The objective of this study was to identify novel genes for resistance to LB from diploid *Solanum tuberosum* L. Andigenum Group (StAG) cultivar accessions. Using comparative analysis with a edgeR bioconductor package for differential expression analysis of transcriptomes, two of these accessions with contrasting levels of resistance to LB were analyzed using digital gene expression data. As a result, various differentially expressed genes (*P* ≤ 0.0001, Log_2_FC ≥ 2, FDR < 0.001) were noted. The combination of transcriptomic analysis provided 303 candidate genes that are overexpressed and underexpressed, thereby giving high resistance to LB. The functional analysis showed differential expression of *R* genes and their corresponding proteins related to disease resistance, NBS-LRR domain proteins, and specific disease resistance proteins. Comparative analysis of specific tissue transcriptomes in resistant and susceptible genotypes can be used for rapidly identifying candidate *R* genes, thus adding novel genes from diploid StAG cultivar accessions for host plant resistance to *P. infestans* in potato.

## Introduction

The oomycete *Phytophthora infestans* (Mont.) de Bary (Peronosporaceae) causes late blight (LB) in potato and other crops in the family Solanaceae. *Phytophthora infestans* is one of the most devastating pathogens worldwide and instigated the tragic Irish famine in the 19th century (Fry et al., 2015). It remains a challenge due to its rapid adaptation to climate change (Wu et al., 2020), early appearances, and its ability to cope with high temperatures (Lehsten et al., 2017; Lurwanu et al., 2021) and ultraviolet radiation (Wu et al., 2019). In Europe, this pathogen accounts for at least US$ 6 billion per year of losses in potato harvests (Haverkort et al., 2016). More than 2,000 MT of fungicide was used to control LB in the USA in 2001 (Magarey et al., 2019). In the Peruvian and Bolivian Andes, which is the primary center of the origin and diversity of the potato crop, control of LB is difficult for poor farmers, who also lack rural extension services. The *P. infestans* population from Peru includes the lineages EC-1, US-1, US-1, PE-3, PE-5, PE-6 and PE-7(Lindqvist-Kreuze et al., 2020). The EC-1 clonal lineage, which shows a complex virulence, is dominant in this country and is characterized by isolates with resistance to metalaxyl. Native potato germplasm are found in high frequency where its crop wild relatives thrive and released cultivars are grown (Lindqvist-Kreuze et al., 2020) including diploid *Solanum tuberosum* L. Andigenum Group (StAG) accessions (formerly known as *S. goniocalyx* Juz. & Bukasov [Ovchinnikova et al., 2011], but it is a synonym no longer accepted), whose center of origin and distribution ranges from central Peru to northern Bolivia. Preventative application of fungicides is being used to control LB. However, their extensive use poses detrimental risks to human health and to the environment (Jiang et al., 2018). Identifying *Solanum* sources of resistance to LB and introducing them into the elite cultigen pool through crossbreeding could become an environmentally benign alternative to fungicides (Enciso-Maldonado et al., 2022).

Research on potato gene expression for LB resistance using different methods (Northern and Southern blots) allows for studying a few genes individually. Next-generation sequencing technology (NGS) (UCLA, 2017) has driven an increase in genetic and genomic research based on DNA or RNA sequences (RNAseq). The deciphering of the potato genome sequences (Xu et al., 2011) enabled the assembling of a reference genome and became a database for differential expression research, thereby promoting the use of methods based on RNAseq (Li et al., 2012; Cevallos Hidalgo, 2015). Using this technology, the leaflet transcriptome of tetraploid *S. tuberosum* ‘Yungay’ when interacting with *P. infestans* (POX-107 and POX-067 strains) was elucidated (Izarra and Lindqvist-Kreuze, 2016). Likewise, high differential expression of genes was noticed in tubers of transgenic plants of tetraploid *S. tuberosum* ‘Russet Burbank’ after performing RNAseq analysis of their tubers interacting with the pathogen *P. infestans* at 48 hours after inoculation (hai) (Gao and Bradeen, 2016). High differential expression at 15 hai was also noticed in tetraploid *S. tuberosum* ‘Bintje’ when interacting with isolates 80029, 88133, and T30-4 of *P. infestans* (Avrova et al., 2003). The same results related to high differential expression at 48 hai under different environments were reported elsewhere (Wang et al., 2005; Ali et al., 2014; Stefańczyk et al., 2017).

Differential expression research with RNAseq in cultivated native potatoes inoculated with *P. infestans* was undertaken with diploid *S. phureja* Juz. & Bukasov [a synonym not accepted of StAG (Ovchinnikova et al., 2011)] (Evers et al., 2006; Massa et al., 2013). Nonetheless, RNAseq research on the other cultivated diploid StAG has not been pursued until this study, the main goal of which was to identify new candidate genes for resistance to *P. infestans* in two transcriptomes differentially expressed in the leaf, using accessions of two diploid-resistant and susceptible StAG cultivars.

## Materials and methods

### Plant materials

Accessions of two diploid (2*n* = 2*x* = 24 chromosomes) *Solanum tuberosum* L. Andigenum Group cultivars that are resistant and susceptible [‘Wira Pasña’ (CIP 704270)] and ‘Sumaq Perqa’ (CIP 703777), respectively] to *P. infestans* from Peru (Pérez et al., 2014; De la Cruz et al., 2020) were used in this study. The International Potato Center (CIP, Lima, Perú) provided the *in vitro* plantlets for this research. They were micro-propagated to increase the number of plantlets by tissue culture using Murashigue and Skoog media and environmental conditions according to Espinoza et al. (1989) and subsequently transplanted into pots with a moss:sand:agricultural soil substrate (2:1:2 v/v) for growth and phenotyping in a greenhouse of the CIP.

### Phenotyping of potato landraces to *Phytophthora infestans*

**Figure 1:**
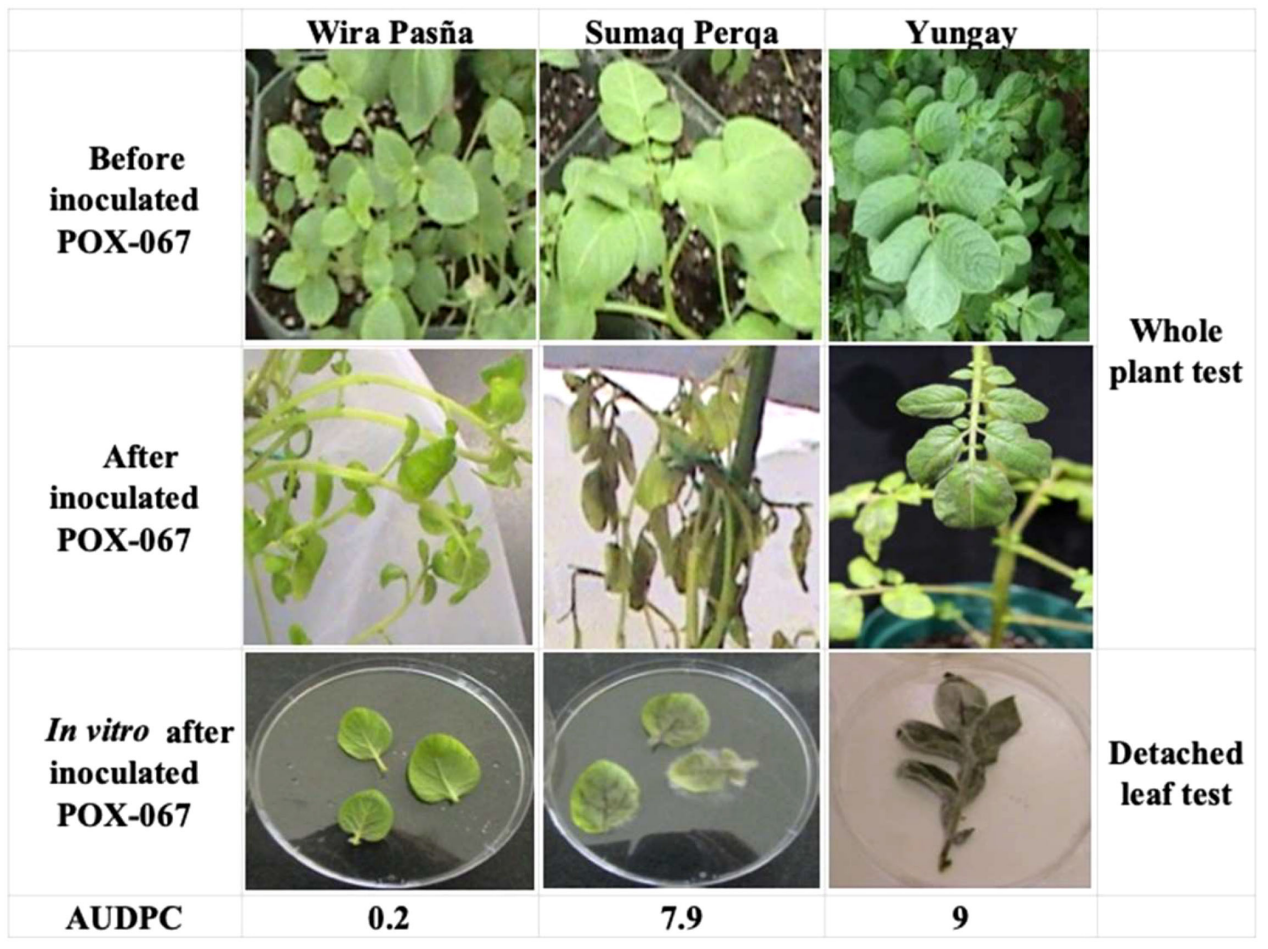
Phenotypic response of diploid *S. tuberosum* cultivar accessions ‘Wira Pasña’ (CIP 704270) and ‘Sumaq Perqa’ (CIP 703777) inoculated with *P. infestans* POX-067. Whole plant of ‘Wira Pasña’ and ‘Sumaq Perqa’ before and after 6 days after inoculation (dai). Detached leaf test in Petri dishes after 6 (dai), the tetraploid *S. tuberosum* ‘Yungay’ was used as a susceptible control. AUDPC = area under disease progress curve.

The *P. infestans* isolate POX-67, which belongs to the clonal lineage EC-1 isolated from Oxapampa (Perú) (Pérez et al., 2014; Izarra and Lindqvist-Kreuze, 2016), previously stored in liquid nitrogen was propagated in potato slices according to an established protocol (Vleeshouwers et al., 1999). Plants of the resistant and susceptible accessions were inoculated before flowering with a concentration of 3,000 sporangia/ml using two methods, detached leaves and whole plants (Forbes et al., 2014; Pérez et al., 2014; Chang-Kee, 2016), to confirm their response to the pathogen (**Figure 1**). For the first method, three leaflets per accession were detached from the middle part of 6-week-old potato plants or before flowering initiation. Leaflets were placed in the lids of inverted Petri dishes with 1.5% water agar in the base and inoculated by placing one 20 µl drop of inoculum containing 3x10^3^ sporangia/ml on the midrib, thus producing one lesion per leaflet. Inoculated leaflets were incubated at 18 ˚C with a 14 h light/day cycle for 5 days after that the percentage of foliage area that is infected by *P. infestans* was determined visually according to Forbes et al. (2014). In the second method for the whole-plant assays, three 6-week-old plants, or before flowering initiation of each accession, were inoculated with a sporangial suspension of 40 ml of inoculum containing 3x10^3^ sporangia/ml until run-off (Eshraghi et al., 2011). After inoculation, the plants were incubated at 16–18 ˚C with a relative humidity of 90% for 7 days. Severity (%) was evaluated once as described by Forbes et al. (2014).

Late blight symptoms caused by *P. infestans* were evaluated in the host potato cultivar using the detached leaf assay. The percentage of diseased leaf area was determined visually according to Forbes et al. (2014) every 48 hai for 6 days. In whole plants, with the severity data (%), the area under the disease progress curve (AUDPC) was calculated (Forbes et al., 2014). The accessions were also assessed through whole plant inoculation, as a second definitive test, which included the tetraploid (2*n* = 4*x* = 48 chromosomes) *Solanum tuberosum* ‘Yungay’ (CIP 720064) as a susceptible control (Forbes et al., 2014; Pérez et al., 2014; Izarra and Lindqvist-Kreuze, 2016). This is the most popular potato cultivar in Peru and is grown in 22% of the total potato planting area (Pradel et al., 2017).

### RNAseq

The transcriptomes were from two leaf sampling times in each cultivar. At 0 hours they were inoculated with water without the pathogen and 48 hai with 3,000 sporangia/ml of *P. infestans* strain POX-67. The experiment included three replications labeled as R48A, R48B, R48C for 48 hai and R0D, R0E, R0F for 0 hai in the resistant cultivar, as well as S48G, S48H, S48I for 48 hai and S0J, S0K, S0L for 0 hai for the susceptible cultivar. In total, 12 libraries were analyzed. *Solanum tuberosum* ‘Wira Pasña’ and ‘Sumaq Perqa’ do not have strictly the same genome because they have evolved into two native Andean cultivars, and they produce two different transcriptomes in each accession. The differential expression test for the comparison of the transcriptome was always from the resistant to the susceptible (R/S) to separate the host plant resistance genes to *P. infestans* only in ‘Wira Pasña’ without the effect of the accessions. Hence, the transcriptome comparisons were between the resistant and susceptible cultivars stimulated by the pathogen at 48 hai (R48/S48); without the stimulus of the pathogen at 0 hai (R0/S0); and by the differential, i.e., (R48/S48) versus (R0/S0) to only obtain the genes differentially expressed by the stimulation of the pathogen without the accession effect in ‘Wira Pasña’.

### RNA extraction and sequencing

Immediately after extracting the leaf samples, they were introduced to liquid nitrogen and transferred to the laboratory, where they were preserved in an ultra-cold chamber (-80°C). The RNA extraction of the 12 leaf samples of both accessions was done in the laboratory of the Genomics Unit of the Universidad Peruana Cayetano Heredia (UPCH, Lima, Perú). The extraction protocol used was pre-tested and standardized (Espinoza, 2017). Leaf tissue (1 to 2 g) was crushed using a mortar and pestle with liquid nitrogen. Thereafter, Tri^®^Reagent (Sigma) (1ml/100g of tissue) and chloroform (200ul/1ml of Tri^®^Reagent) were used to continue the centrifugation series without breaking the cold chain (4°C) and to avoid RNA degradation. The precipitation of the “pellet” was achieved with isopropanol and washing with 75% ethanol. The samples were treated with a DNA-freeTM Kit (Ambion, USA) to clean contaminating DNA, ensuring optimal quality and quantity of the total RNA. The quality of total RNAs was confirmed through the absorbance ratio on a Thermo Scientific Spectrophotometer NanoDrop 2000, the RNA Integrity Number (RIN) on an Agilent 2000 Bioanalyzer (Plant RNA Nano Chip, Agilent), and the absence of smear.

The concentration of total RNA extracted ranged from 93.13 to 468.01 ng/ul, with an average of 297.63 ng/ul, in an average extraction volume of 69.66 ul. The purity values ​​of the RNA evaluated by spectrophotometry were on average 2.14 (absorbance A260/A280) and 2.23 (absorbance A260/A230). The RIN integrity values ranged from 7.7 to 8.6, with an average of 8.13. The results of the amount of total extracted RNA (ng/ul) and the absorbance and integrity of the RNA of the 12 samples are given in **Supplementary Table 1**.

Sequencing was performed on the Illumina Hi-Seq 2500 platform, with Q30 (i.e., below 1/1000 of sequenced nucleotides) and a sequencing accuracy of 99.9%. The size of the reading fragments was 150 bp. Twelve cDNA paired-ends (PE) libraries were constructed. This process was achieved through the service of NovoGene Corporation Inc. (California, USA, https://en.novogene.com/).

### Bioinformatic analysis of RNAseq data

The bioinformatic analysis (Gollery, 2006) was performed using the server and platform in a Linux environment (Carreño, 2017). FastQC (Leggett et al., 2013) (http://www.bioinformatics.babraham.ac.uk/projects/fastqc/) was used to assess the quality of the sequences considering only libraries with Q ≥ 33. Trimmomatix (Bolger et al., 2014) (https://github.com/kbaseapps/kb_trimmomatic) was used to clean the sequences of the Illumina adapters, considering a score of 33 and filtering the libraries with a minimum length of 145 bp in the reads. STAR (Dobin and Gingeras, 2015) was used for the mapping of the libraries to the reference genome of *S. phureja*, which was annotated by the Potato Genome Sequencing Consortium (PGSC) DM v4.03 (Xu et al., 2011). The *.sam files were converted to *.bam using SAMtools (http://www.htslib.org) (Li et al., 2009). SUBREAD (featureCounts) facilitated the quantification of the levels of expression of transcripts between treatments, obtaining a “count table” with the number of reads mapped to each segment of the genome (“transcripts”) and their corresponding values ​​in count-per-million (CPM), which were further used to detect differentially expressed genes (DEGs) among resistant and susceptible accessions.

The predictive analysis of DEGs when comparing the two transcriptomes of both cultivar accessions in response to stress with *P. infestans* was performed using the bioconductor package *edgeR* in the Rstudio environment. The input (table count data) was extracted to the R environment through *read.delim* and principal component analysis (PCA) of the 12 libraries with unfiltered data was performed with the *DGEList* and *plotMDS* packages. The filtering was performed with CPM rather than filtering the counts directly. CPMs of differentially expressed transcripts were then normalized using the *cpm* and *calcNormFactors* packages. The model was defined considering the experimental design using the *model.matrix* and *estimateDisp* packages so that the dispersion of the transcripts was only due to the effect of the treatments. The adjustment of the values was made to the quasi-likelihood (QL) F-tests, since the number of biological replicates was below five (Law et al., 2018), using the *glmQLFTest* and *TopTags* packages. Differentially expressed transcripts or candidate genes for host plant resistance were considered if they had a greater difference in abundance with Log_2_FC ≥ 2 up-expressed and Log_2_FC ≤ -2 down-expressed, at a probability of significance below or equal to 0.0001, and with a probability of false positive readings below 0.001 with the Benjamini-Hochberg method. “complexHeatmap” and “ggplot2” were used to make the smear, volcano, and heatmap plots. Likewise, the software Venny 2.1 (Oliveros, 2016) was used to organize the DEG sets in a Venn Euler diagram.

Functional analysis of the over and underexpressed transcripts was performed separately. The gene ontology (GO) and the enrichment of DEGs in metabolic pathways noted in the Kyoto Encyclopedia of Genes and Genomes (KEGG) was facilitated by the g:Profiler software (Raudvere et al., 2019) (https://biit.cs.ut.ee/gprofiler/gost) using the database of *S. tuberosum* PGSC_GENE, providing the identifiers (IDs) of the genes and their corresponding significant value (padj < 0.05) over and underexpressed peptides. To calculate the padj, the Benjamini-Hochberg FDR (false discovery rate) method was used, using g:Profiler and g:GOSt (functional profiling), with the user threshold at 0.05.

## Results

### Phenotyping of diploid StAG cultivar accessions

This experiment used whole plants of two diploid StAG cultivar accessions, namely the LB-resistant ‘Wira Pasña’ (CIP 704270) and the susceptible ‘Sumaq Perqa’ (CIP 703777), which were previously evaluated using a detached leaf assay. The susceptible tetraploid *S. tuberosum* ‘Yungay’ was used as a control (**Figure 1**). The resulting area under the disease progress curve (AUDPC) was 0.2 and 7.9 respectively for the resistant and susceptible diploid StAG cultivar accessions and 9 for ‘Yungay’.

### Sequencing

The results of the Illumina sequencing, in the *.fasta files of the 12 libraries, were reported considering paired reads (PE). The readings read in direction 5’ to 3’, “forward”, were identified by adding _1 to the library code, those that were read in direction 3’ to 5’, “reverse”, were identified by adding _2. Therefore, 24 libraries resulted whose sequencing yields (**Supplementary Table 2**) resulted in the range of 22´426,271 to 32´315,289 reads per library with an average of 27´370,780. All of them exhibited good quality (Q30) sequencing as analyzed with FastQC. In total, 656´898,720 readings were obtained in the 12 paired libraries. Approximately 94% of the readings (on average 26´270,256 readings per bookstore) “survived” the “cleaning” with *Trimmomatic* (**Supplementary Table 3**).

### Mapping of reads to the reference genome

**Table 1** lists the results of the alignment of the reads to the indexed reference genome (*S. tuberosum* Soltub.3.0.dna.toplevel.fa.gz). Approximately 92% of the total reads were mapped, of which 88% of the reads were at single sites, 4% of the reads were at more than one site, and 8% of the reads were not mapped to the reference genome. There were 40,336 transcripts (i.e., total genes that are annotated) in the 12 libraries. After filtering the counts with *edgeR*, 19,666 aligned transcripts were obtained without distorting the modeling in all repetitions and in at least one treatment.

**Table 1:** Number of reads and percentage of reads, mapped to the *Solanum tuberosum* Soltub.3.0.dna.toplevel.fa.gz reference genome, from 12 StAG diploid libraries.

Sample Name	Total reads	Total mapped		Multiple mapped		Uniquely mapped		Unmapped
		N	%			N	%	(%)
R48A	47,398,026	43,353,448	91.47	1,654,926	3.49	41,698,522	87.98	8.53
R48B	51,218,548	46,818,700	91.41	1,847,371	3.61	44,971,329	87.80	8.59
R48C	57,910,202	53,204,824	91.87	1,976,333	3.41	51,228,491	88.46	8.13
S48G	59,893,568	54,666,092	91.27	2,045,387	3.42	52,620,705	87.86	8.73
S48H	54,708,012	49,917,835	91.24	1,964,538	3.59	47,953,297	87.65	8.76
S48I	72,287,734	66,225,093	91.61	2,615,413	3.62	63,609,680	88.00	3.89
R0D	41,740,904	38,532,626	92.31	1,607,250	3.85	36,925,376	88.46	7.69
R0E	52,261,774	48,450,142	92.71	2,062,379	3.95	46,387,763	88.76	7.29
R0F	63,342,836	58,632,077	92.56	2,716,340	4.29	55,915,737	88.27	7.44
S0J	42,225,972	39,194,192	92.82	1,901,763	4.50	37,292,429	88.32	7.18
S0K	52,638,960	48,959,396	93.01	3,788,764	7.20	45,170,632	85.81	6.99
S0L	58,355,444	53,596,973	91.85	2,381,674	4.08	51,215,299	87.76	8.15
Total	653,981,980	601,551,398		26,562,138		574,989,260		

### Differential expression analysis

**Figure 2:**
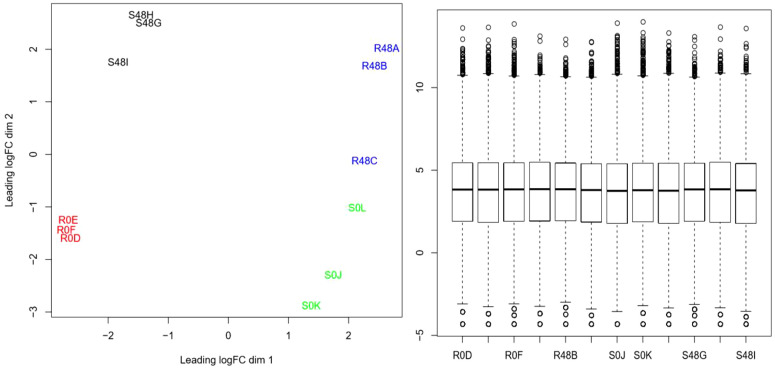
Grouping of 12 libraries (40,336 transcripts) by principal component analysis in resistant and susceptible diploid StAG cultivar accessions. The left graph shows the trend of four groups with opposite and contrasting locations. The grouping trends were in opposite locations for the samples of the resistant cultivar at 0 hours after inoculation (hai) (R0D, R0E, and R0F in red) and at 48 hai (R48A, R48B, and R48C in blue), thereby confirming the proper handling of the samples. The samples of the susceptible cultivar at 0 hai (S0J, S0K, and S0L in green) and those at 48 hai (S48G, S48H, and S48I in black) were in opposite groupings. The right graph shows the boxplot representation of the 12 libraries (19,666 genes) normalized with *edgeR* software in Rstudio.

Principal component analysis (PCA) was performed on all expressed transcripts (40,336 transcripts) from the 12 libraries (**Figure 2**, left, all treatments away from the center). The result of the normalization of the 12 libraries is shown in the boxplot of **Figure 2** (right), in which the averages of each library were standardized and normalized with homogeneous distributions and averages of the CPM values.

### First Analysis between resistant and susceptible transcriptomes without the stimulus of the pathogen at 0 hai (R0/S0)

**Figure 3:**
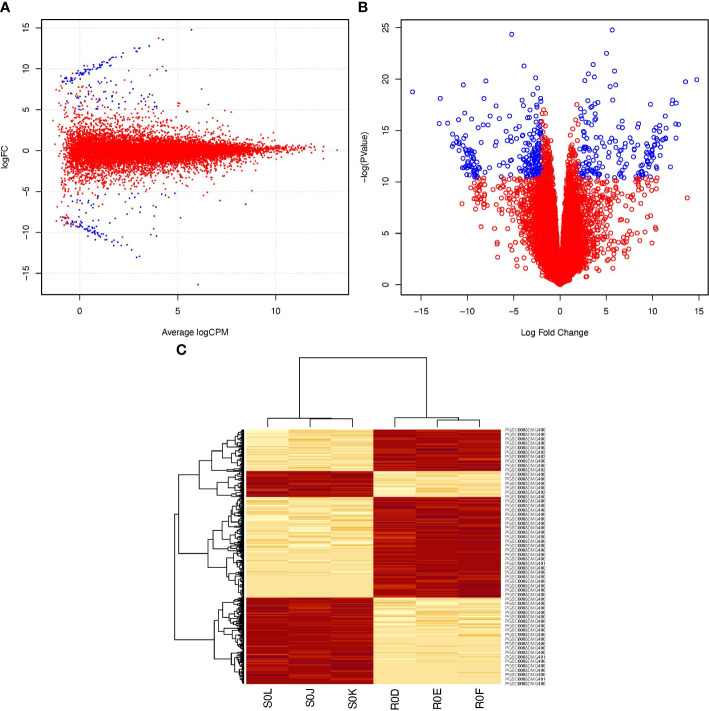
Differential expression profile of 400 genes between resistant *S. tuberosum* ‘Wira Pasña’ versus susceptible *S. tuberosum* ‘Sumaq Perqa’, without stimulation of the pathogen *P. infestans* at 0 hours after inoculation (hai). In Box **(A)** (smear plot) and Box **(B)** (volcano plot), the genes represented in blue were overexpressed (Log_2_FC >+2) and underexpressed (Log_2_FC < -2), respectively. In box **(C)**, four clusters are observed in the horizontal heatmap, of which the two red clusters include the overexpressed genes in ‘Wira Pasña’ and two other groups of different genes that were overexpressed in ‘Sumaq Perqa’. The vertical heatmap shows the grouping trend of the 400 genes of the resistant ‘Wira Pasña´) in two vertical clusters with different intensities (heat) of expression in the six libraries at 0 hai, of which three correspond to ‘Wira Pasña’ (R0D, R0E, and R0F) and another three to ‘Sumaq Perqa’ (S0J, S0K, and S0L). The intensity of expression of the clustered genes is related to the intensity of color. The more intense the red, the more overexpressed (> E) the gene, and the less intense, the less expressed the genes, until reaching white, when they are repressed (< E).

The transcriptomes of the resistant ‘Wira Pasña’ and the susceptible ‘Sumaq Perqa’ were differentially expressed at 0 hai. In total, 400 genes in the transcriptome of ‘Wira Pasña’ (**Supplementary Table 5**; https://docs.google.com/spreadsheets/d/1paCEZkQXS6kSgNEnTb5p9r6FJJimoFYF/edit?usp=sharing&ouid=110777050509080518982&rtpof=true&sd=true) had an accession (A) effect. These genes are the ones that transcriptomically differentiate this resistant cultivar from the susceptible ‘Sumaq Perqa’ when they are not stimulated by the pathogen (0 hai). They were used in the following analysis as baseline differentially expressed genes (DEGs). The distribution of said differentially expressed genes is shown in **Figure 3**.

### Second analysis between resistant and susceptible transcriptomes stimulated by the pathogen (P) at 48 hai (R48/S48)

**Figure 4:**
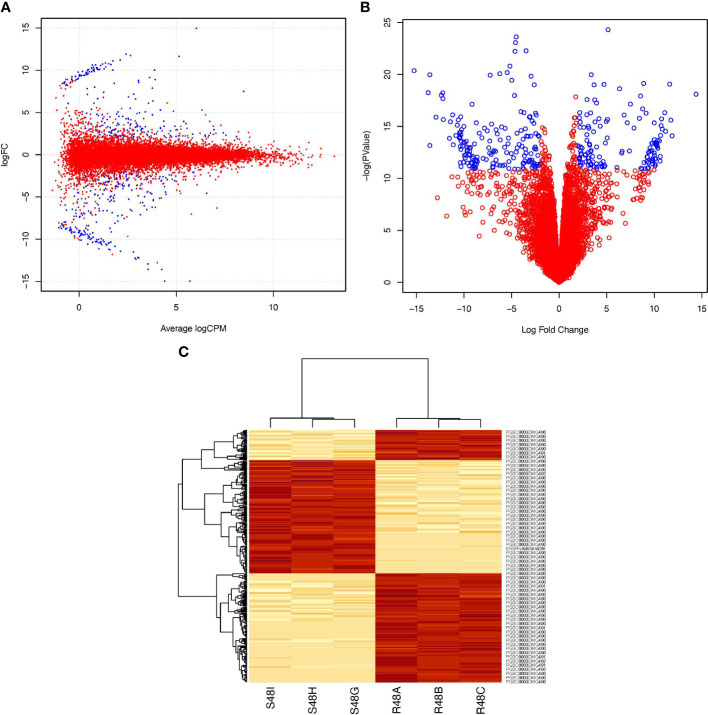
Differential expression profile of 303 genes between the resistant ‘Wira Pasña’, the combined effect of ‘Sumaq Perqa’ and ‘Wira Pasña’, plus the effect of *P. infestans pathogen* at 48 hours after inoculation (hai). In Box **(A)** (smear plot) and Box **(B)** (volcano plot) the genes represented in blue were overexpressed (Log_2_FC > +2) and underexpressed (Log_2_FC > -2), respectively. In Panel **(C)**, the horizontal heatmap shows three clusters, including a red cluster of overexpressed genes in ‘Wira Pasña’ and two other red groups of different genes that were overexpressed in ‘Sumaq Perqa’. The vertical heatmap shows the grouping trend of the 303 differential genes in two clusters with different intensities (heat) of expression in the libraries of ‘Sumaq Perqa’ (S48G, S48H, and S48I) and ‘Wira Pasña’ (R48A, R48B, and R48C) at 48 hai. In the clusters, there was a difference between a group of genes that were overexpressed and two groups that were underexpressed in the resistant cultivar ‘Wira Pasña’ because of more pathogenic accessions (A+P). The intensity of expression of the clustered genes is related to the intensity of color. The more intense the red, the more overexpressed (> E) the gene, and the less intense, the less expressed the gene, until reaching white (< E).

There were 303 genes differentially expressed 48 hai in the transcriptome of the resistant cultivar after being stimulated with *P. infestans* (**Supplementary Table 4**; https://docs.google.com/spreadsheets/d/1LGJqSFDtgcoRl65pg8ei94tLq1pUbZqF/edit?usp=sharing&ouid=110777050509080518982&rtpof=true&sd=true). These genes transcriptomically differentiate ‘Wira Pasña’ from ‘Sumaq Perqa’, in addition to the effect of the accessions. **Figure 4** shows the distribution of the differentially overexpressed and underexpressed genes (Log_2_FC >2 and <-2, respectively, and FDR ≤ 0.001).

### Third analysis based on previous differential transcriptomes (R48/S48 versus R0/S0)

**Figure 5:**
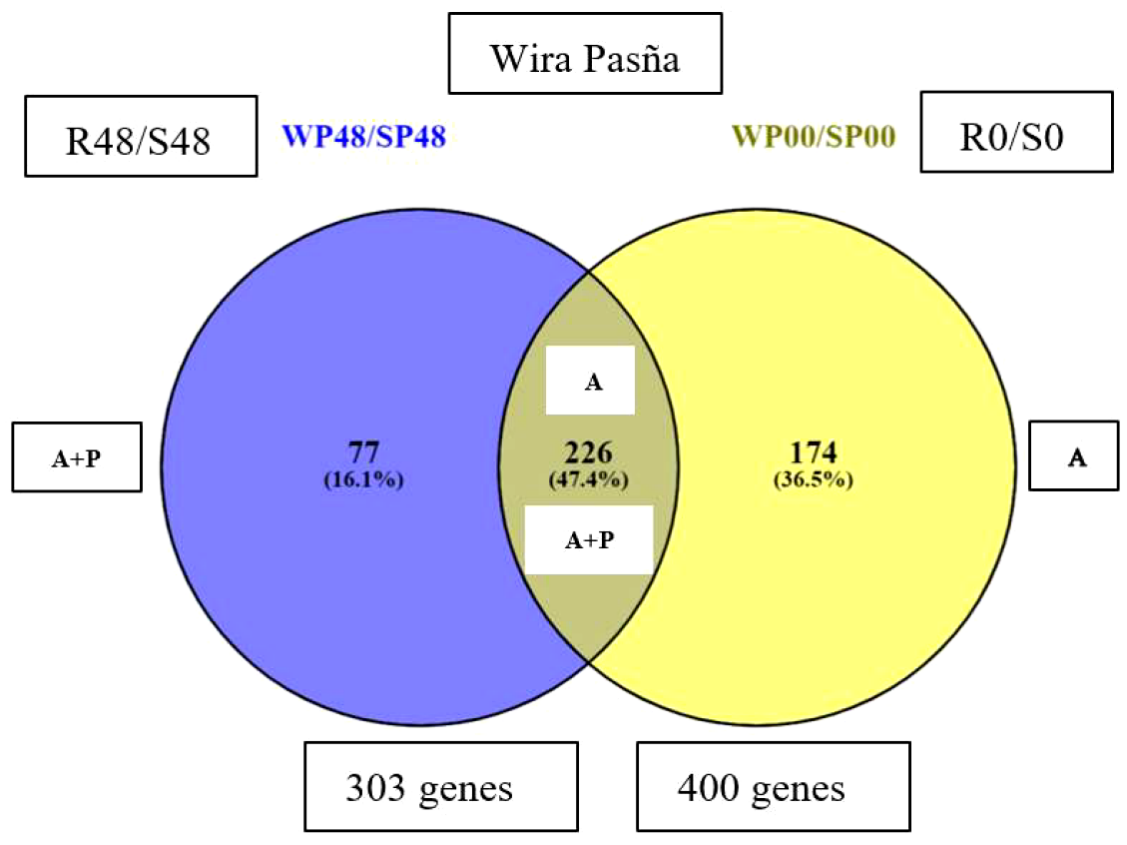
A Venn-Euler diagram showing, in the blue subset, that there were 77 genes expressed in the resistant ‘Wira Pasña’ by the combined effect of accessions plus treatment (A+P) at 48 hours after inoculation (hai), while in the yellow subset, there were 174 genes expressed in ‘Wira Pasña’ by the accessions effect (A). At the intersection, there are 226 genes that were expressed by the combined effect (A+P) in addition to the accessions effect (A) and were expressed in different directions.

There were 400 differential genes obtained from the previous basal differential quantification between the resistant cultivar against the susceptible cultivar at 0 hai (WP0/SP0), and another 303 differential genes noticed at 48 hai (WP48/SP48). They are included in a Venn-Euler diagram (**Figure 5**), which shows that 77 unique genes were differentially expressed in the resistant ‘Wira Pasña’, after being stimulated 48 hai by the combined accession effect and that of the pathogen (A+P).

**Figure 6:**
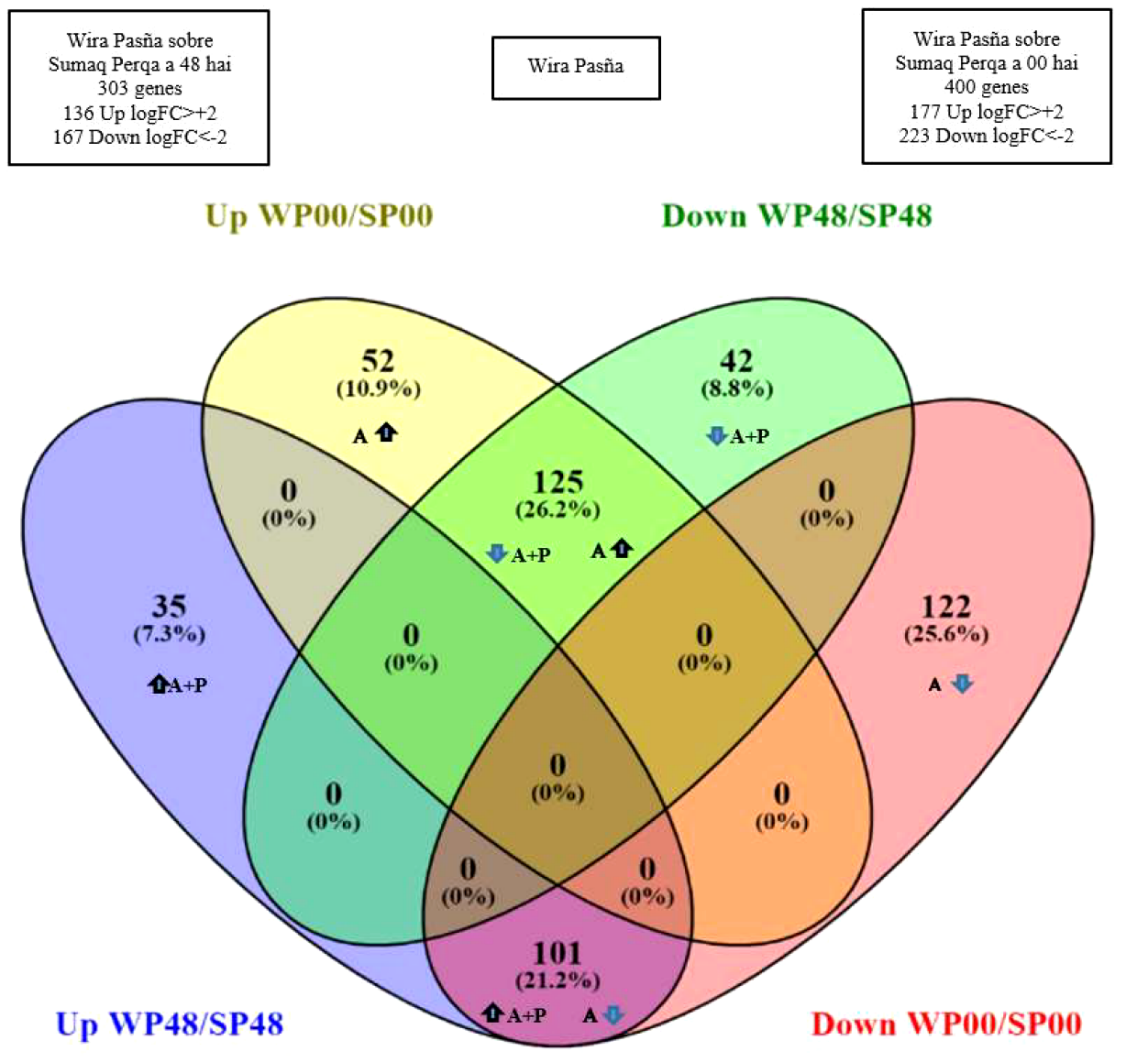
Venn-Euler diagram representing the genes of resistant cultivar ‘Wira Pasña’. There were 35 and 52 genes upregulated by the combined effect (A+P) at 48 hours after inoculation (hai) and accession effect (A) at 0 hai, respectively (blue and yellow subsets respectively), of which only the subgroup of 35 genes (blue subset) are overexpressed by the combined effect (A+P). There were 42 and 122 genes downregulated by A+P at 48 hai and by A at 0 hai, respectively (green and red subsets, respectively), of which only the subgroup of 122 genes (subset red) was underexpressed by A. In addition, 226 genes were differentials (subsets intersections 101 and 125 genes) that were expressed in opposite directions (arrows) when stimulated by A+P and A.

The combined effect of accessions plus pathogen (A+P) at 48 hai stimulated 303 genes to be differentially expressed in ‘Wira Pasña’, of which 136 genes (**Figure 6**, blue set) were overexpressed (Log_2_FC > +2, FDR ≤ 0.001). From this group, 35 genes were only upregulated (blue subset). In addition, 167 genes (green set) were underexpressed (Log_2_FC > -2, FDR ≤ 0.001). In this last group, 42 genes were only underexpressed (green subset) in the resistant accession.

There were 400 genes (**Figure 6**) differentially expressed in the ‘Wira Pasña’ at 0 hai, of which 177 genes (yellow set) were overexpressed (Log_2_FC > +2, FDR ≤ 0.001). From that group, only 52 genes (yellow subset) were only overexpressed due to the accession effect (A). In addition, 223 genes (red set) were underexpressed (Log_2_FC > -2, FDR ≤ 0.001) and from this last group, 122 genes were only underexpressed (red subset) due to A.

In the resistant ‘Wira Pasña’, 226 genes with differential expression were quantified (subsets with 101 and 125 genes, **Figure 6**). They were expressed in the opposite direction because of the accession (A) and accessions plus pathogen (A+P) effects. The 101 differential genes were overexpressed due to A+P and these same ones were underexpressed due to A. The other 125 genes were overexpressed due to A and were underexpressed by stimuli of A+P at 48 hai.

The relationship of genes over and underexpressed in the transcriptome of ‘Wira Pasña’, considering both A+P and A, are given in **Supplementary Table 4**. There were 303 genes related to host plant resistance to *P. infestans* (**Figure 6**), of which 136 were overexpressed genes (35 + 101 genes) and 167 were underexpressed genes (42 + 125 genes) (Log_2_FC > 2 and <-2, respectively and FDR ≤ 0.001).

### Gene ontology enrichment analysis

**Figure 7:**
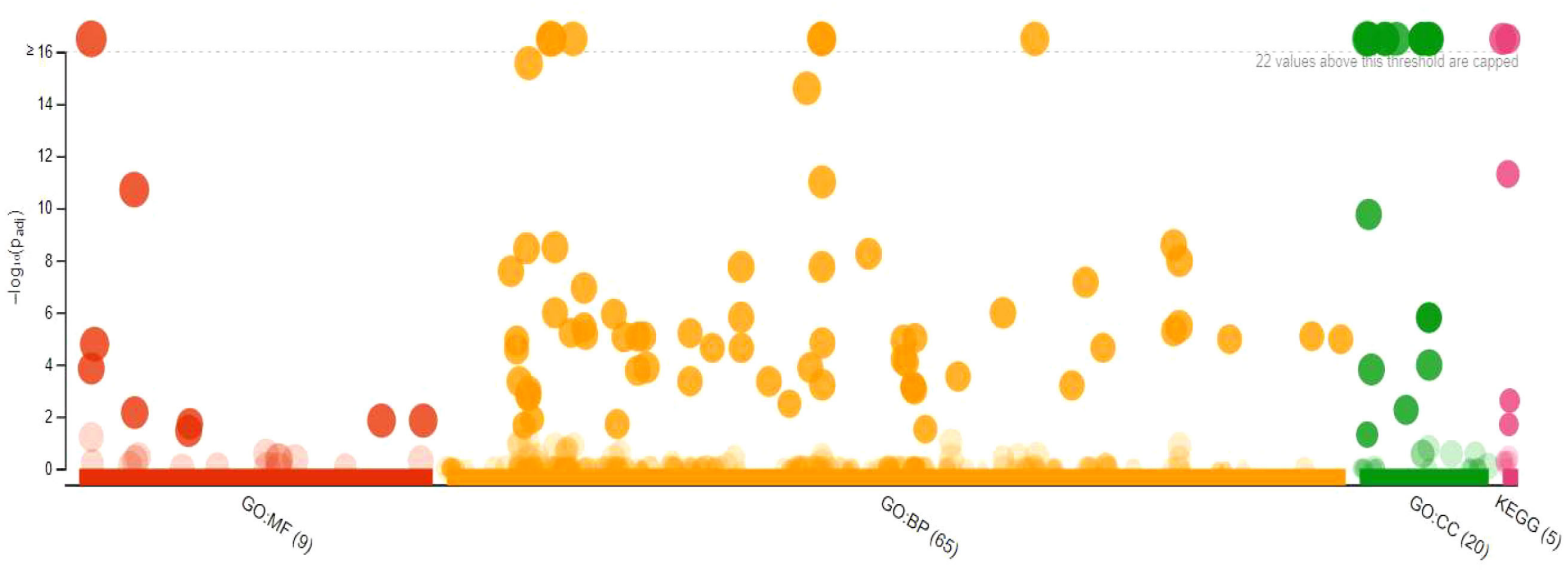
Manhattan plot of 167 underexpressed differentially expressed genes (DEGs) from diploid *Solanum tuberosum* ‘Wira Pasña’ (CIP-704270) after gene ontology enrichment analysis using g:Profiler.

**Figure 8:**
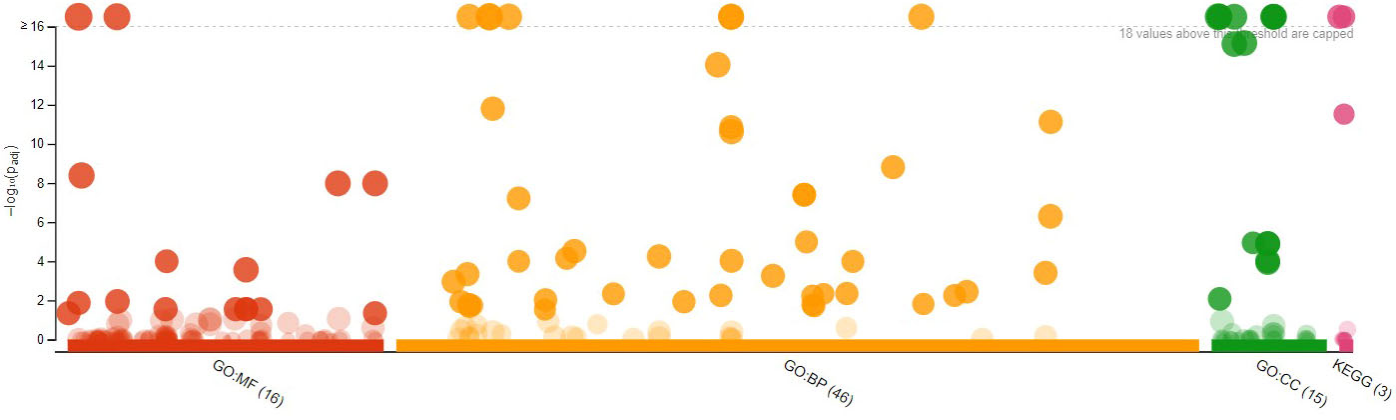
Manhattan Plot of 136 overexpressed differentially expressed genes (DEGs) from diploid *Solanum tuberosum* ‘Wira Pasña’ (CIP-704270) after gene ontology enrichment analysis using g:Profiler.

The GO analysis with GO term and adjusted *P*-values from 303 DEGs is given **Supplementary Table 6** (https://docs.google.com/spreadsheets/d/1s9WK4_gRhnq9b8bv5rrgjLg8eE1DXyrr/edit?usp=sharing&ouid=110777050509080518982&rtpof=true&sd=true). Of these, 167 underexpressed DEGs are shown in **Figure 7**, while the 136 overexpressed DEGs of resistant ‘Wira Pasña’ are given in **Figure 8**. The 167 underexpressed DEGs were enriched in the three main categories of the GO. **Figure 7** shows the nine genes enriched for molecular function (GO.MF), 65 genes corresponding to biological process terms (GO.BP), and 20 related to cellular components (GP.CC).

The binding function (GO:0003674) had the highest significance (padj. 1.937x10^-11)^ in the molecular function category, followed by the functions of catalytic activity (GO:0003824) and binding of nucleic acids (GO:0003676) with pad. 1.668 × 10^-5^ and 1.432 × 10^-4^, respectively. The other molecular functions were non-significant. For the biological process category, the metabolic process (GO:0008152) and the cellular process (GO: 0009987) had the highest significance (both with padj, 1.006 × 10^-31)^). The metabolic processes of organic substances (GO:0071704), primary metabolic processes (GO:0044238), cellular metabolic processes (GO:0044237), metabolic processes of nitrogenous compounds (GO:0006807), macromolecular metabolic processes (GO:0043170), and metabolic processes of cellular macromolecules (GO:0044260) had high significance (padj range: 1.547 × 10^-26^ to 9.836 × 10^-12^). The other biological processes were not significant. Membrane components (GO: 0005623) had high significance (padj. 4.119 × 10^-30^) in the cellular components category, followed by the intracellular component (GO:0005622), intrinsic components of the membrane, organelles (GO:0031224), and organelle membrane-bound intracellular (GO:0043231) with padj. ranging from 2.178 × 10^-26^ to 2.425 × 10^-22^. The other cellular components were not significant.

The 136 overexpressed DEGs were enriched in the three main categories of the GO. **Figure 8** shows the 16 genes enriched for molecular function (GO.MF), of which 46 genes were related to biological process terms (GO.BP) and 15 to cellular components (GO.CC).

The binding function (GO:0005488) had the highest significance (padj. 1.937 × 10^-17)^ in the molecular function category, followed by the functions of catalytic activity (GO:0003824), binding of heterocyclic components (GO:1901363), and components of organic cycles (GO:0097159) with padj. ranging from 4.131 × 10^-9^ to 1.047 × 10^-8,^ and the hydrolase activity function (GO:0016787) with padj. of 1.011 × 10 ^-4.^ The other molecular functions were not significant. The metabolic process (GO:0008152) and the cellular process (GO:0009987) were the most significant (padj, 1.574x10^-39^ and 1.202x10^-32^, respectively). in the biological process category. The cellular processes (GO:0009987), metabolic processes of organic substances (GO:0071704), primary metabolic processes (GO:0044238), cellular metabolic processes (GO:0044237), metabolic processes of nitrogenous compounds (GO:0006807), metabolic processes of macromolecules (GO:0043170), and biosynthesis processes (GO:0009058) had high significance with their Padj ranging from 1.202 × 10^-32^ to 1.587 × 10^-12^. The other biological processes were not significant. Cellular components (GO:0044464) and the intracellular component (GO:0005622) had high significance (padj. 1.703 × 10^-36^ and 1.359 × 10^-31^, respectively) in the cellular components category, followed by intracellular components (GO:0044424), membrane (GO:0016020), and membrane intrinsic components (GO:0031224) with padj. ranging from 1.703 × 10^-30^ and 1.359 × 10^-16^. The other cellular components were not significant.

### Kyoto encyclopedia of genes and genomes metabolic pathway enrichment analysis

**Figure 9:**
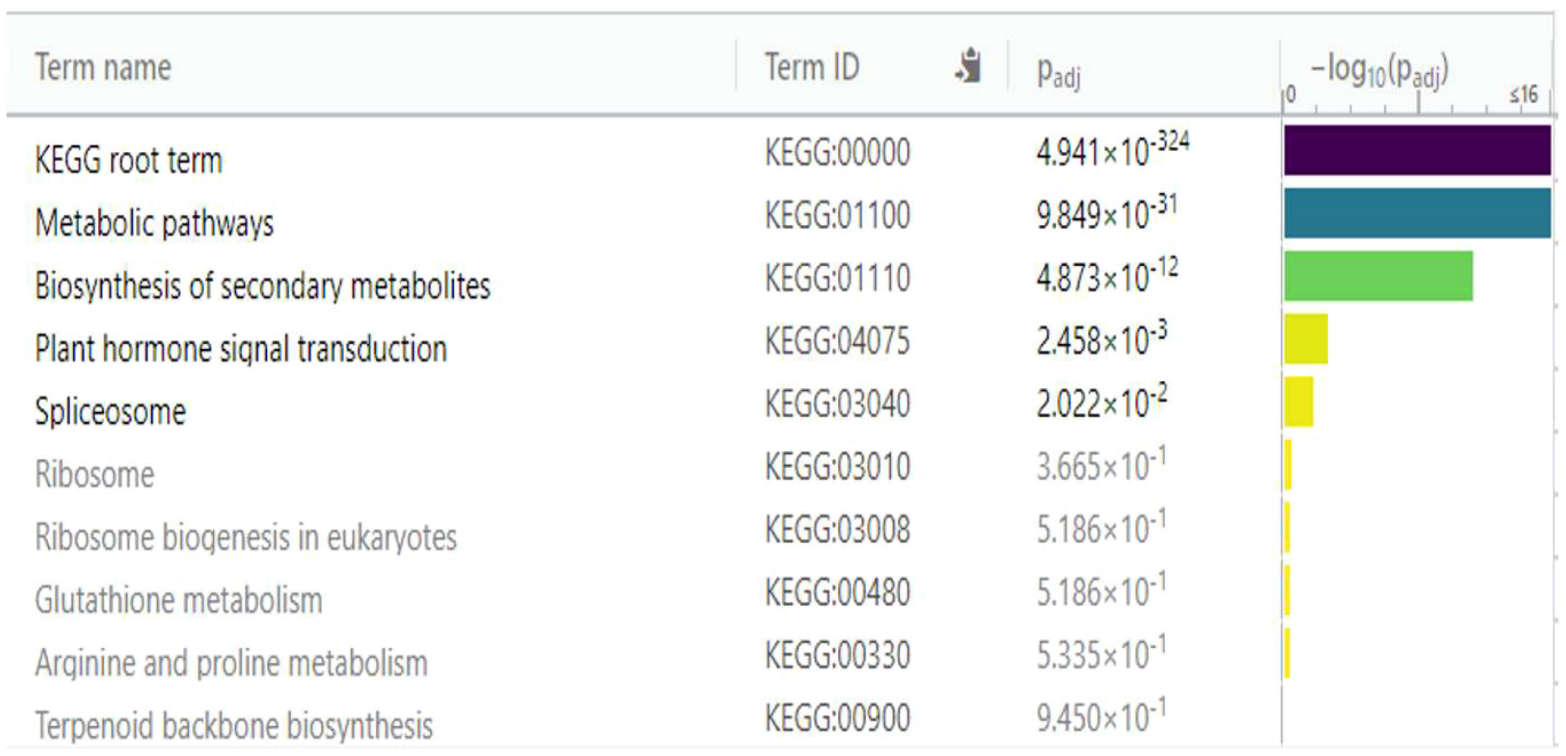
Enrichment analysis of 167 underexpressed differentially expressed genes (DEGs) in diploid *Solanum tuberosum* ‘Wira Pasña’ (CIP-704270), compared using the KEGG database. g:Profiler was used with significance at padj < 0.05.

**Figure 10:**
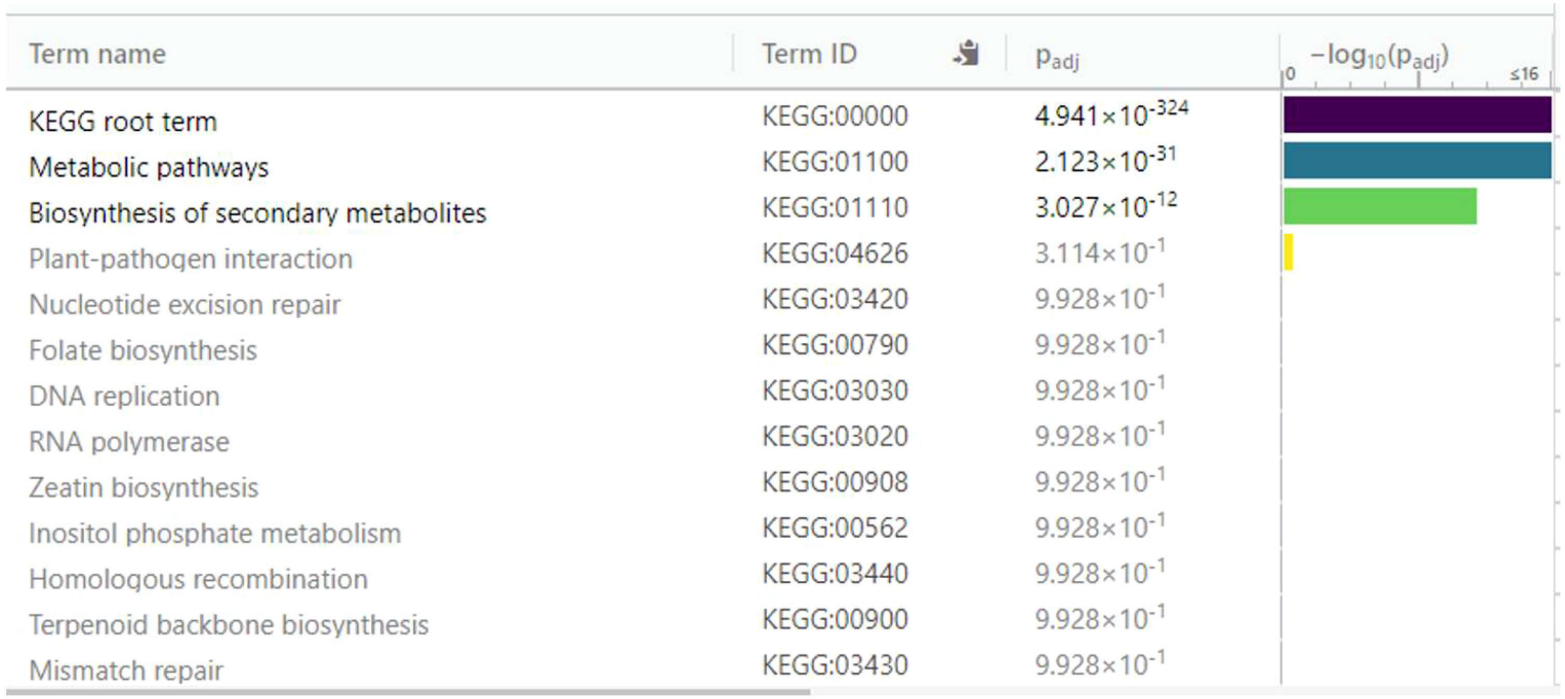
Enrichment analysis of 136 differentially expressed genes (DEGs) overexpressed in diploid *Solanum tuberosum* ‘Wira Pasña’ (CIP-704270) compared using the KEGG database. g:Profiler was used with significance at Padj < 0.05.

KEGG serves as a basic platform for the systematic analysis of gene function in terms of networks or metabolic pathways of gene products (Kanehisa, 2019) (Kanehisa and Goto, 2000) (Kanehisa et al., 2023). It was used to identify the biosynthetic pathways that are active in the resistant ‘Wira Pasña’ after being infected by the *P. infestans* isolate POX-067. The first analysis was for the underexpressed DEGs (**Figure 9**), and thereafter for the overexpressed ones (**Figure 10**).

The metabolic pathways (KEGG:01100) and the secondary metabolite biosynthesis pathway (KEGG:01110) had a high significance (padj. of 9.849 × 10^-31^ and 4.873 × 10^-12,^ respectively, considering the annotation of 167 underexpressed DEGs in the KEGG database. Likewise, plant hormone transduction signal pathways (KEGG:04075) and the spliceosome pathway (KEGG:03040) were significant (padj of 2.458 × 10^-3^ and 2.022 × 10^-2^, respectively). The other routes were not significant.

Of the 136 overexpressed DEGs that were annotated to the KEGG database, two main enriched pathways, namely, the metabolic pathway (KEGG:01100) and the secondary metabolite biosynthesis pathway (KEGG:01110), were highly significant (padj. 2,123 × 10^-31^ and 3,027 × 10^-12^, respectively). The plant-pathogen interaction pathway (KEGG:04626) with a padj of 3.114 × 10^-1^ was also found, although it was not significant, as well as the other remaining routes.

### Candidate R genes for resistance of StAG accessions against *P. infestans* isolate POX-067

A total of 20 genes were found from the enrichment of the 167 underexpressed genes and the 136 overexpressed DEGs at 48 hai (**Table 2**). They are related to resistance to pathogens in general, of which 10 of these genes were underexpressed, namely, one gene for virus resistance (*PGSC0003DMG402016602*), another gene for bacteria (*PGSC0003DMG401012062*), four genes for protein resistance NBS-LRR (*PGSC0003DMG401007871*, *PGSC0003DMG400007605*, *PGSC0003DMG400002217*, and *PGSC0003DMG400007870*), two genes (*PGSC0003DMG400016372* and *PGSC0003DMG400018462*) for protein conferring disease resistance, the *PGSC0003DMG400005471* gene of Receptors Like Kinases (RLK) type, and the *PGSC0003DMG402024222* gene for BRASSINOSTEROID INSENSITIVE 1-associated receptor kinase. Among these 10 genes underexpressed at 0 hai, four belong to the NBS-LRR gene family, and one is an RLK type, in addition to the gene for “BRASSINOSTEROID INSENSITIVE 1-associated receptor kinase”.

**Table 2:** List of 20 candidate genes related to host plant resistance in diploid *S. tuberosum* ‘Wira Pasña’ (CIP-704270) to *P. infestans* isolate POX-067, at 48 hours after inoculation (hai).

Underexpressed genes					
Gene ID		Description	Log_2_FC 00hai	Log_2_FC 48hai	FDR
*PGSC0003DMG402016602*		Tospovirus resistance protein C	8.20	-11.12	1 × 10^-4^
*PGSC0003DMG401007871*		NBS-LRR protein	2.22	-2.35	1 × 10^-5^
*PGSC0003DMG400007605*		NBS-LRR protein	6.03	-6.95	5 × 10^-5^
*PGSC0003DMG400002217*		NBS-LRR resistance protein	5.57	-5.67	5 × 10^-5^
*PGSC0003DMG400007870*		NBS-LRR protein	5.28	-5.93	7 × 10^-5^
*PGSC0003DMG400016372*		Resistance protein PSH-RGH7	6.70	-7.64	2 × 10^-5^
*PGSC0003DMG401012062*		Bacterial spot disease resistance protein 4	8.39	-8.48	7 × 10^-5^
*PGSC0003DMG400018462*		Disease resistance protein	3.77	-4.26	5 × 10^-6^
*PGSC0003DMG400005471*		RLK_6-phosphoglucono-lactonase	3.71	-2.38	4 × 10^-5^
*PGSC0003DMG402024222*		BRASSINOSTEROID INSENSITIVE 1-associated receptor kinase	3.75	-4.67	1 × 10^-5^
Overexpressed genes					
Gene ID		Description	Log_2_FC 00hai	Log_2_FC 48hai	FDR
*PGSC0003DMG400008596*		Cc-nbs-lrr resistance protein	-8.29	8.47	3 × 10^-5^
*PGSC0003DMG400025547*		Late blight resistance protein	-11.59	11.59	8 × 10^-4^
*PGSC0003DMG400003380*		Late blight resistance protein homolog R1A-4	-3.88	4.08	1 × 10^-6^
*PGSC0003DMG400005590*		Disease resistance protein	-7.54	7.32	2 × 10^-4^
*PGSC0003DMG400002427*		Bacterial spot disease resistance protein 4	-4.02	3.03	1 × 10^-4^
*PGSC0003DMG400031277*		F-box domain-containing protein	-10.0	10.01	2 × 10^-4^
*PGSC0003DMG400036554*		F-box protein	-9.48	9.48	1 × 10^-4^
*PGSC0003DMG400031279*		F-box domain-containing protein	-10.15	10.15	1 × 10^-4^
*PGSC0003DMG400008593*		Flavonoid glucoyltransferase UGT73E2	-4.25	5.90	1 × 10^-4^
*PGSC0003DMG400017234*		Auxin-induced protein 5NG4	-2.49	2.90	1 × 10^-4^

It is important to highlight that of the 103 genes (of the 303 DEGs) with unknown function from ‘Wira Pasña’, 55 of them were underexpressed and the remaining 48 genes were overexpressed in the presence of P. infestans at 48 hai. They were annotated using g:Profiler and the PGSC_GENES database.

The 10 genes overexpressed for host plant resistance to *P. infestans* in diploid StAG accessions (**Table 2**) were one gene (*PGSC0003DMG400008596*) from the NBS-LRR family of resistance proteins, two genes (*PGSC0003DMG400025547* and *PGSC0003DMG400003380*) for resistance proteins against late blight, another gene (*PGSC0003DMG400005590*) for protein disease resistance at large, a gene (*PGSC0003DMG400002427*) encoding proteina4 resistance against bacterial disease, three genes (*PGSC0003DMG400031277*, *PGSC0003DMG400036554*, and *PGSC0003DMG400031279*) for translation factors, a gene (*PGSC0003DMG400008593*) for flavonoid glucoyltransferase UGT73E2, and the *PGSC0003DMG400017234* gene for auxin-induced protein 5NG4. Among these 10 genes, the NBS-LRR gene and the two specific genes for resistance proteins against late blight stand out. They were underexpressed at 0 hai when challenged by the pathogen *P. infestans* but they were overexpressed at 48 hai.

## Discussion

As noted by previous research, when determining the phenotype of an accession or genotype, the concentration of inoculum, type strain (virulence, aggressiveness), inoculation method, and environment interaction are very important for the performance of the experiment (Ali et al., 2014; Gao and Bradeen, 2016; Izarra and Lindqvist-Kreuze, 2016: Stefańczyk et al., 2017). Considering the background of the POX-067 strain, this experiment used examined and referenced concentrations, as well as known inoculation methodology (Izarra and Lindqvist-Kreuze, 2016). The time of inoculation (before flowering) and sampling (48 hai) used in this study were based on available literature (Wang et al., 2004; Evers et al., 2005; Riva Romero, 2010; Monsalve-Fonnegra et al., 2012; Ali et al., 2014; Stefańczyk et al., 2017), which indicated high differential expression after evaluating *S. phureja* inoculated with *P. infestans* between 36 and 48 hai.

The interactions between the *S. tuberosum* accessions of cultivars ‘Wira Pasña’ and ‘Sumaq Perqa’ with the pathogen *P. infestans* (**Figure 1**) confirmed them as resistant and susceptible, respectively, as previously known (Pérez et al., 2014). *Solanum tuberosum* ‘Yungay’ was susceptible (Wulff et al., 2007) and is frequently used as a control for the evaluation of host plant resistance to LB (Lindqvist-Kreuze et al., 2014; Jiang et al., 2018), as well as for pathogenicity research (Lindqvist-Kreuze et al., 2020). This cultivar made it possible to validate the screening results.

In the present work, paired reads have been used to improve the accuracy of the mapping to the reference genome. The mapping alignment of unique sequences was 88%, which is higher than previously known (Gao and Bradeen, 2016). Approximately 92% of reads landing in the reference genome are DNA sequences that correspond to the species, thus indicating that the reference genome used in this study is of good quality. The remaining 8% of unmapped sequences suggests that the gene space of the reference genome used in this study does not contain this group of genes specific to diploid accessions due to species differences. The reference genome is more closely related to *S.phureja* (Xu et al., 2011).

The location and distribution of the 12 bookcases in the PCA coordinate plane were opposite (**Figure 2**), which indicates that the resistant and susceptible cultivars with and without *P. infestans* inoculation had an adequate variance within and between each factor. Normalization is used to correct for differences in the sizes of raw data libraries so that this does not influence the calculations and differential analyses. Some authors (Li et al., 2012) proposed a normalization method that assumes a Poisson count model and estimates the sequencing depth. However, we used *edgeR*, which models and fits a negative binomial distribution. To normalize, the *edgeR* software, such as *DESeq*, calculates the ratios between the gene counts in each sample, and the geometric mean of the gene counts in all samples, while the library size is estimated as the median of the ratios between genes (Anders and Huber, 2010). Different available results (Dillies et al., 2013; Finotello et al., 2014) indicate that the *edgeR* and *DESeq* methods are the most suitable for normalizing the size of the library (Lovén et al., 2012; Liu et al., 2015; Rapaport et al., 2015) because they use direct counts for calculating the mean and the variance. In this study the sample size was small (3 samples per condition), and the data were normalized (**Figure 2**) and analyzed with *edgeR.* When the sample size is small (2 to 5 samples per condition), the best results are generally obtained with *DESeq* and *edgeR*. (Soneson and Delorenzi, 2013). The correlation analyses of the expressed DEGs were based on the statistical power of edgeR, demonstrated in the heat maps and Manhattan plots, and are as valid as the weighted correlation networks analysis (WGCNA) used by Esposito et al. (2019); Esposito et al. (2021)

In this study of the transcriptome of StAG cultivar accessions stressed with *P. infestans* at 48 hai, there were significantly underexpressed genes in the presence of the pathogen, while the secondary metabolite biosynthesis pathways were highly significant for both the underexpressed and overexpressed genes. These results were similar to those observed in a transgenic *S. tuberosum* transcriptome at 0, 6, and 24 hai in the foliage (Gao and Bradeen, 2016), as well as in transcriptomic research in *Citrus* stressed with *P. parasitica* (Naveed et al., 2019). It is important to highlight that there were 103 genes with unknown functions among the 303 DEGs. They are probably genes of ‘Wira Pasña’, of which 55 of them were underexpressed and the other 48 were overexpressed in the presence of *P. infestans* at 48 hai.

The oomycete *P. infestans* is an extraordinarily virulent and adaptable pathogen, secreting several effector proteins that modulate the host’s innate immunity (Vleeshouwers et al., 2008). All known avirulence genes belong to the RXLR effector classes, and encode secretory proteins with the RXLR motif for translocation into cells (Vleeshouwers et al., 2011). The *P. infestans POX 067* isolate used in the present study expresses the avirulence genes *Avr8* and *Avr9* (Izarra and Lindqvist-Kreuze, 2016), thereby generating effector proteins that were possibly identified in the first instance by the overexpressed genes at 0 hai with isolate *POX 067* (**Table 2**, highlighting 10 overexpressed genes at 0 hai) that then activate overexpressed *R* genes at 48 hai in a second instance (**Table 2**, highlighting 10 genes with high log_2_ FC). This was demonstrated by Lin et al. (2022), with the widely conserved *Phytophthora* RXLR-WY effector, which was first recognized through the *Rpi-amr3* gene that activated the expression of resistance genes. These findings suggest a new way to redeploy *R* genes that recognize known effectors against *P. infestans*.

We found 303 genes (**Supplementary Table 4**), of which 136 overexpressed genes related to LB resistance. Considering the difference in expression level (Log_2_FC) from 0 to 48 hai, in addition to selecting only genes that code for resistance, 20 DEGs were found to give host plant resistance to *P. infestans* in accessions of diploid cultivars of S*. tuberosum* Andigenum Group. Of these, 10 genes (**Table 2**) that were overexpressed at 0 hai (log_2_FC, range, 2.22 to 8.39), were underexpressed at 48 hai (log_2_FC ranging from -2.35 to -11.12) in the presence of the pathogen. In contrast, another 10 genes that were underexpressed at 0 hai (log_2_FC ranging from -2.49 to -11.59) were overexpressed in the presence of the pathogen at 48 hai (log_2_FC ranging from 2.90 to 11.59). Similar results were noted after evaluating the gene expression profile of tetraploid S*. tuberosum* SD20 for its resistance against *P. infestans* (Yang et al., 2019). Due to their differential expressions, GO analysis and KEEG enrichment reported 10 overexpressed genes closely related to LB resistance: PGSC0003DMG400008596 (Cc-nbs-lrr resistance protein), PGSC0003DMG400025547 (late blight resistance protein), PGSC0003DMG400003380 (Late blight resistance protein homolog R1A-4), PGSC0003DMG400005590 (disease resistance protein), PGSC0003DMG400002427 (bacterial spot disease resistance protein 4), PGSC0003DMG400031277 (F-box domain-containing protein), PGSC0003DMG400036554 (F-box protein), PGSC0003DMG400031279 (F-box domain-containing protein), PGSC0003DMG400008593 (flavonoid glucoyltransferase UGT73E2), and PGSC0003DMG400017234 (auxin-induced protein 5NG4).

Plants have evolved efficient defense mechanisms based on a molecular immunity system through *R* genes that allow them to resist different pathogens including bacteria, fungi, oomycetes, viruses, and nematodes. Most *R* genes (approximately 80%) encode nucleotide binding sites (NBS) and comprise three domains that allow them to be classified into TIR-NBS-LRR (TNL), CC-NBS-LRR (CNL), or RPW8-NBSLRR (RNL), of which the C-terminal LRR domain exhibits high diversity and has been associated with pathogen recognition (Qian et al., 2017). At the beginning of the infection with *P. infestans*, underexpressed genes increased, especially NBS-LRR (nucleotide binding site, leucine-rich repeats), which could indicate that at the beginning, many underexpressed genes are required to generate resistance in the late stages of infection (Yang et al., 2018). The probable reason for this is that many of the resistance genes are constitutively expressed. Thus, there were four NBS-LRR genes in ‘Wira Pasña’ that were underexpressed at the beginning of the infection. Indeed, in this study, the resistant cultivar overexpressed a CC-NBS-LRR gene at 48 hai, thereby highlighting its classification as a candidate gene together with two other LB protein-specific resistance genes to *P. infestans*.

The genes of the NBS-LRR domain are related to R proteins (Lozano et al., 2012). In *S. tuberosum* the highest number of NBS-LRR genes are on chromosomes 4 and 11 (15% of mapped genes), with a lower number on chromosome 3 (1%) (Jupe et al., 2013). *R* proteins are responsible for the recognition of pathogen proteins called effectors, which allow the host hypersensitivity reaction, thereby preventing infection. *PR* (pathogenesis-related proteins) genes are distributed into 17 families (Shi et al., 2019). They have been described, isolated, and characterized in *S. phureja* in relation to host plant resistance to *P. infestans* in the field (Evers et al., 2005; Evers et al., 2006). *PR-1* gene *mRNA* has also been evaluated in resistant and susceptible potato cultivars to confirm the role of *PR-1* for host plant resistance to *P. infestans* in the field. In the present study, we report six *R* genes, five from the NBS-LRR domain and one from the RLK domain, that were stimulated by *P. infestans*. Of the five, four genes from the NBS-LRR domain were underexpressed at 48 hai and one from the CC-NBS domain (*PGSC0003DMG400008596*) was overexpressed at 48 hai (**Table 2**). These genes seem to allow the hypersensitivity in accessions of diploid cultivars of S*. tuberosum* Andigenum Group, thus preventing infection by *P. infestans*. The *PGSC0003DMG400005471* gene of the RLK type noted in this study confirms previous results in tetraploid potato (Yang et al., 2018). Hence, RLKs are important genes for plant-pathogen recognition receptors in the early stages of the attack of *P. infestans* in potato.

It was observed in ‘Wira Pasña’ that an enzyme related to MAPK (a brassinosteroid associated with kinase receptors) was overexpressed at the beginning of the infection. Brassinosteroids are considered one of the most important steroid hormones with many roles in plants, e.g., adaptation of plants to different stress factors and successful stress tolerance (Khan et al., 2022). In the early stages of infection by *P. infestans*, DEGs mainly encode defense enzymes to increase their expression, thus triggering a phosphorylation cascade through protein kinase (MAPK) that directly suppresses the pathogen as the first line of defense (Yang et al., 2018). RLKs that are in the plasmatic membrane in plants, positively regulate the innate immunity of the plant (Yang et al., 2018). In this study, one RLK gene was greatly overexpressed at 48 hai, thus suggesting that these RLKs are important recognition receptors in the early stages of host plant resistance to LB.

Transcription factors play a crucial role in resistant plants by modulating the transcription of resistance-related genes for the binding of specific DNA sequences to their promoter regions (Li et al., 2017). F-box proteins constitute a family of diverse transcription factors in prokaryotes and eukaryotes and regulate the cardinal biological processes in plants including growth and development, cellular protein degradation, and response to biotic or abiotic stress (Zhao et al., 2017), in addition to being involved in the biosynthesis of anthocyanins (Zhang et al., 2020). In potato, 11 transcription factors are strongly upregulated, including WRKY1, WRKY3, and WRKY5, thereby suggesting positive regulation of these WRKYs in the resistance response to *P. infestans* infection (Yang et al., 2018). ‘Wira Pasña’ had an overexpression of three genes of F-box proteins at 48 hai, which suggests that their functional role also occurs under such biotic stress. This result strongly supports the selection of these genes for host plant resistance to *P. infestans*. In addition, further functional research on the F-box genes will be necessary to explore their roles in the response to *P. infestans*-induced stress in potato.

UDP-glycose flavonoid glycosyl-transferase (UFGT) catalyzes metabolites closely related to the biosynthesis of flavonoids and anthocyanins, which are stimulated by stressors such as low temperatures (10°C or less) (Yu et al., 2017). In this study, there was an overexpression at 48 hai of the flavonoid *UGT73E2* gene, which agrees with a previous finding where the temperature for the progress of *P. infestans* infection was low (Yu et al., 2017). It has also been found that an increased expression of DEGs at 24 hai encodes defense enzymes and disease-related proteins, including flavonoid-3-hydroxylase (Yang et al., 2018). Similarly, in ‘Wira Pasña’, there was increased expression of the flavonoid *UGT73E2* gene at 48 hai due to the inductive effect of the pathogen, thereby triggering phosphorylation cascades, regulating the expression of genes downstream related to resistance proteins against the pathogen.

To date, more than 70 *Rpi* genes have been identified and mapped in 32 *Solanum* species. High-level host plant resistance has been found in several diploid Mexican species, including *Solanum bulbocastanum* Dunal. and *S. pinnatisectum* Dunal., most of which have been derived from wild and tuber-bearing species, four from Peru, but none from the diploid *S. tuberosum* Andigenum Group (Paluchowska et al., 2022). This work is the first report highlighting 20 major *R* genes, including highly expressed *P. infestans* effector detectors at 0 hai and other resistance candidate *R* genes at 48 hai and more than 300 minor genes (**Supplementary Table 6**) related to horizontal and durable resistance. The combined use of *Rpi* genes that recognize the essential and conservative effectors of *P. infestans* and pyramiding *Rpi* genes may assist in achieving long-lasting, broad-spectrum resistance to late blight.

The goal to develop resistance in potato crops to the pathogen may not be easy to accomplish because of the evolution of pathogens to overcome, with time, any resistance conferred by *R* genes (Majeed et al., 2022), thereby suggesting that more systematic approaches are consistently required. They may use wild *Solanum* germplasm sources for resistance genes (Nelson et al., 2018). Although difficult to be accomplished, quantitative host plant resistance conferred by several minor genes seems to be a better strategy for achieving durable host plant resistance to *P. infestans* (Du et al., 2015; Nelson et al., 2018). In this investigation, we found 10 major *R* genes (high Log_2_FC) at 48 hai and more than 300 minor genes (**Supplementary Table 6**) related to quantitative trait loci for late blight resistance in the *S. tuberosum* Andigenum Group transcriptome, thus strengthening our knowledge of resistance induction in potato against the pathogen *P. infestans*. The identification of new sources of genes with durable resistance to *P. infestans* in native potatoes such as the diploid *S. tuberosum* Andigenum Group would contribute to the improvement of this tuber crop.

The *PGSC0003DMG400025547* and *PGSC0003DMG400003380* genes encode host plant resistance to *P. infestans* in diploid StAG accessions. The first gene encodes LB resistance proteins and the other an LB resistance protein homologous to *R1a-4*. These two genes were underexpressed (log_2_FC -11.59 and -3.88, respectively) when diploid StAG accession was not inoculated at 0 hai, but they were overexpressed (log_2_FC 11.59 and 4.08, respectively) at 48 hai by *P. infestans* stimulation. These diploid StAG accession genes could be different from the *Rpi-vnt1 genes* previously known (*PGSC0003DMG400024364* and *PGSC0003DMG400024363*), (van der Vossen et al., 2003), e.g., *Rpi* ancestral genes of the wild potato *S. bulbocastanum* that confer broad resistance to *P. infestans*. In the context of the Peruvian Andes, where the EC-1 clonal lineage of *P. infestans* predominates (Perez et al., 2001), the potential use of the *Rpi-vnt1, Avr-blb1 y Avr-blb2* genes and of the two specific of diploid StAG accessions (PGSC0003DMG400025547 and PGSC0003DMG400003380) facilitates breeding for host plant resistance to *P. infestans* along with the genes for the NBS-LRR proteins found in this study (FDR, range 1 × 10^-4^ to 1 × 10^-6^).

## Conclusion

This leaf transcriptome research was able to identify candidate genes that provide host plant resistance to LB in StAG accessions. There were 303 genes differentially expressed by the pathogen stimulus, of which 10 overexpressed genes were defined as *R* genes for the defense response: PGSC0003DMG400008596, PGSC0003DMG400025547, PGSC0003DMG400003380, PGSC0003DMG400005590, PGSC0003DMG400002427, PGSC0003DMG400031277, PGSC0003DMG400036554, PGSC0003DMG400031279, PGSC0003DMG400008593y, and PGSC0003DMG400017234. Of these, four genes (*PGSC0003DMG400008596*, *PGSC0003DMG400025547*, *PGSC0003DMG400003380*, and *PGSC0003DMG400005590*) encode for NBS-LRR proteins. These results provide further evidence that diploid cultivated potatoes are a source of host plant resistance genes to the pathogen *P. infestans*.

## Data availability statement

The datasets presented in this study can be found in online repositories. The names of the repository/repositories and accession number(s) can be found at: https://www.ncbi.nlm.nih.gov/, Bioproject PRJNA861684 and PRJNA861685.

## Author contributions

GC, conception and design-execution of all stages of the project. RB, project design. WP, design-execution of phenotyping and editing manuscript drafts. EN, Design-execution RNA extraction/preservation, sequencing, and being co-corresponding author. RO, supervision, writing manuscript and editing drafts, and being co-corresponding author. All co-authors read and agreed on the text of the article. All authors contributed to the article and approved the submitted version.
