# Supplementary material for: Foliar transcriptomes reveal candidate genes for late blight resistance in cultivars of diploid potato Solanum tuberosum L. Andigenum Group
Source: Front Plant Sci. 2023 Sep 11;14:1210046. doi: 10.3389/fpls.2023.1210046 (PMC10535101; doi:10.3389/fpls.2023.1210046)

**Supplementary Table 1**: Amount (ng/ul) of RNA extracted from *Solanum tuberosum* L*.* Andigenum Group potato leaf samples, and purity indicators by spectrophotometry through the absorbance coefficients A260/A280 and A260/A230. In addition, RNA Integrity Number (RIN), Aguilent 2000, stressed with *P. infestans* according to experimental design.

| N° | Sample Name | Conc. (ng/μl) | Vol. (μL) | A260/A280 | A260/A230 | RIN |
| --- | --- | --- | --- | --- | --- | --- |
| 1 | R48A | 468.01 | 70 | 2.15 | 2.26 | 7.7 |
| 2 | R48B | 284.83 | 69 | 2.14 | 2.12 | 8.4 |
| 3 | R48C | 384.39 | 70 | 2.12 | 2.37 | 8.2 |
| 4 | R0D | 198.62 | 69 | 2.13 | 2.23 | 8 |
| 5 | R0E | 189.24 | 71 | 2.18 | 2.13 | 8 |
| 6 | R0F | 281.37 | 71 | 2.14 | 2.36 | 8 |
| 7 | S48G | 320.84 | 68 | 2.14 | 2.00 | 8.4 |
| 8 | S48H | 454.08 | 70 | 2.15 | 2.14 | 7.8 |
| 9 | S48I | 93.13 | 71 | 2.11 | 2.29 | 8.4 |
| 10 | S0J | 355.16 | 70 | 2.14 | 2.00 | 8.1 |
| 11 | S0K | 293.59 | 69 | 2.15 | 2.36 | 8.6 |
| 12 | S0L | 248.38 | 68 | 2.13 | 2.23 | 8 |
| Average |  | 297.63 | 69.66 | 2.14 | 2.24 | 7.7 |

**Supplementary Table 2**: Illumina Hiseq 2500 sequencing performance, considering poor reads (PE) (code_1 and code _2).


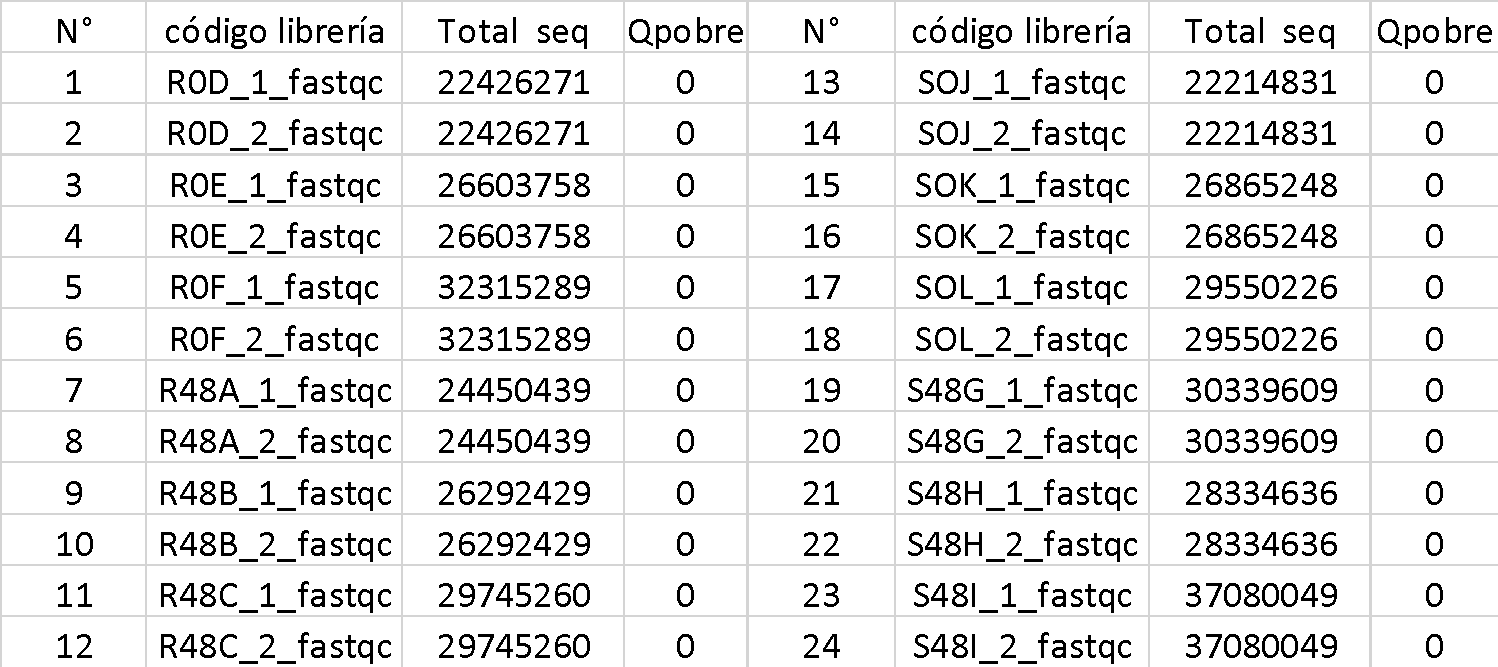


**Supplementary Table 3**: Result of cleaning the reads of the 12 libraries of *Solanum tuberosum* L*.* Andigenum Group using the *Trimomatric* Software, the average survival was 93.66%.

| Library code | Reads Input | Reads output | Survival (%) |
| --- | --- | --- | --- |
| R48A | 24450439 | 22827755 | 93.36 |
| R48B | 26292429 | 24745408 | 94.12 |
| R48C | 29745260 | 279752586 | 94.05 |
| R0D | 22426271 | 20398374 | 90.96 |
| R0E | 26603758 | 24975298 | 93.88 |
| R0F | 32315289 | 30386620 | 94.03 |
| S48G | 30339609 | 28640618 | 94.40 |
| S48H | 28334636 | 26478467 | 93.45 |
| S48I | 37080049 | 34921050 | 94.18 |
| S0J | 22214831 | 20582748 | 92.65 |
| S0K | 26865248 | 25272790 | 94.07 |
| S0L | 29550226 | 28038690 | 94.88 |
| Total | 336218045 | 315243076 |  |
| Average | 28018170.42 | 26270256.33 | 93.66 |
|  |  |  |  |

**Supplementary Table 4**: List of 303 differentially expressed genes in *Solanum tuberosum* L*.* Andigenum Group, resistant accession CIP-704270, Wira Pasña, at 48 hai that confer resistance against *P. infest* POX-067, of which 136 genes are over-expressed (35 +101 genes) and 167 genes are under-expressed (42 +125 genes) in the resistance process. Effect accessions + pathogen (A+P). More information such as the description of the genes, Log2FC, P value or FDR in the table shared through google drive: <https://docs.google.com/spreadsheets/d/1LGJqSFDtgcoRl65pg8ei94tLq1pUbZqF/edit?usp=sharing&ouid=110777050509080518982&rtpof=true&sd=true>

**Supplementary Table 5.** List 400 genes between resistant cultivar ‘Wira Pasña’ versus susceptible cultivar ‘Sumaq Perqa’*,* without stimulation of the pathogen *Phytophthora infestans* at 0 hai (Figure 3C) <https://docs.google.com/spreadsheets/d/1paCEZkQXS6kSgNEnTb5p9r6FJJimoFYF/edit?usp=sharing&ouid=110777050509080518982&rtpof=true&sd=true>)

**Supplementary Table 6.** GO_term_from_303_DEG_ *Solanum tuberosum* L*.* Andigenum Group _48hpi_with *P. infestans* <https://docs.google.com/spreadsheets/d/1s9WK4_gRhnq9b8bv5rrgjLg8eE1DXyrr/edit?usp=sharing&ouid=110777050509080518982&rtpof=true&sd=true>

**Supplementary Figure 1**: Enrichment analysis of 167 under expressed differentially expressed genes (DEGs) in *Solanum tuberosum* L*.* Andigenum Group cultivar ‘Wira Pasña’ (CIP-704270), compared with the KEGG database g:Profiler was used with significance at Padj < 0.05.


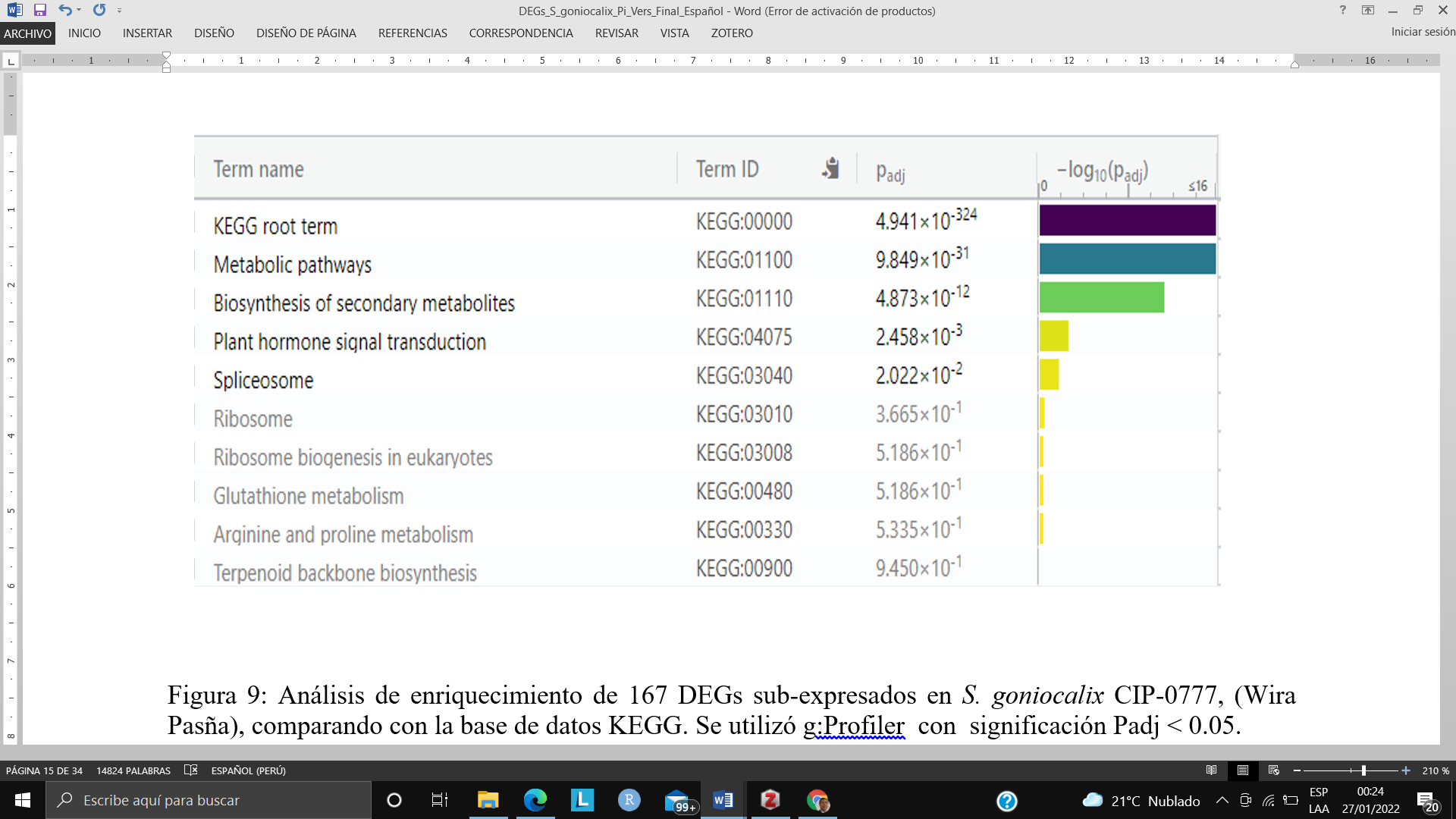


**Supplementary Figure 2**: Enrichment analysis of 136 over expressed differentially expressed genes (DEGs) in *Solanum tuberosum* L*.* Andigenum Group cultivar Wira Pasña (CIP-704270), compared with the KEGG database g:Profiler was used with significance at Padj < 0.05.


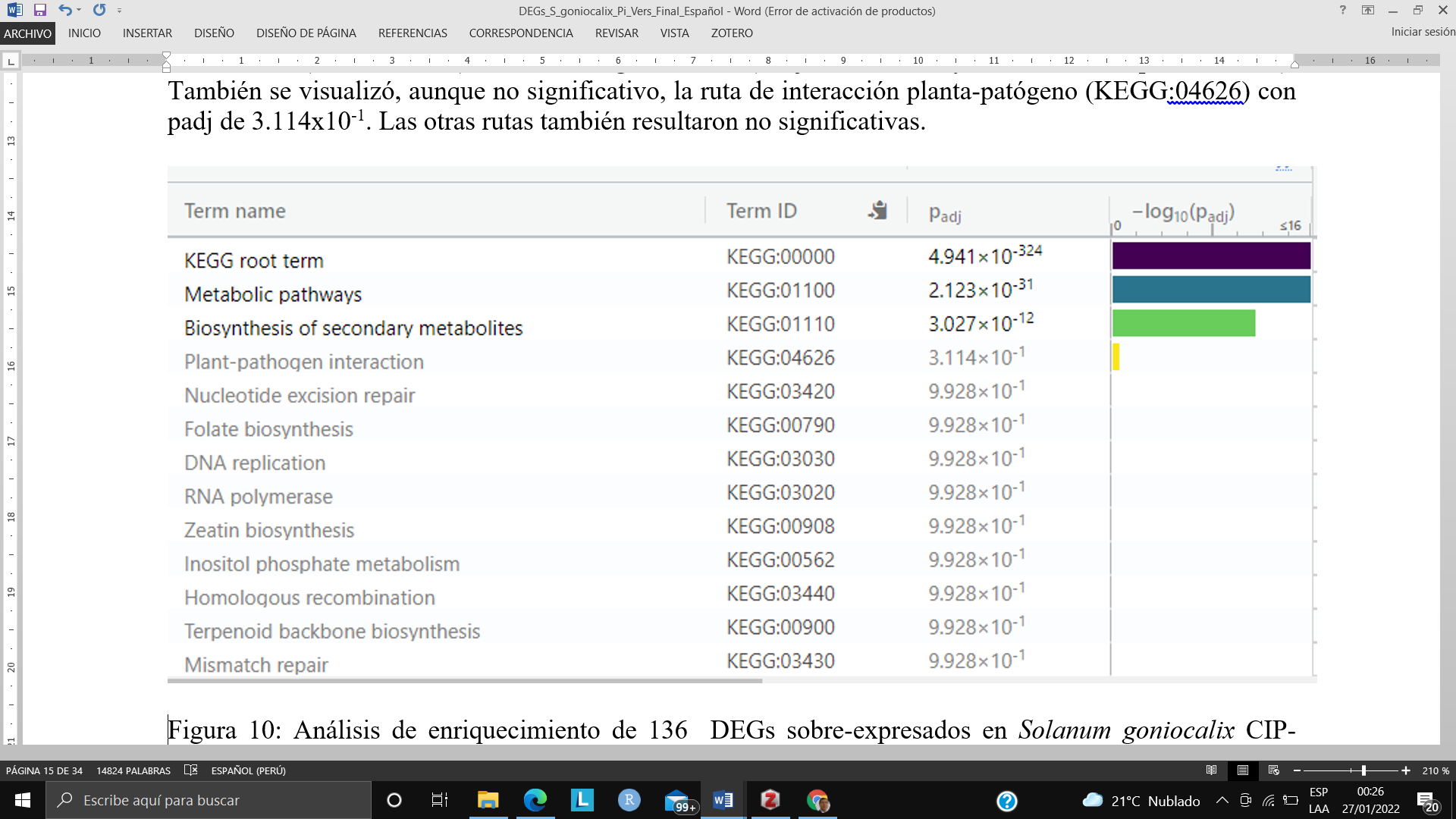

Supplement: Supplementary file 1 [file DataSheet_1.docx]
